# Coexistence of Two Different Thyroid Malignancies: A Collision Phenomenon

**DOI:** 10.7759/cureus.7539

**Published:** 2020-04-04

**Authors:** Reza Pishdad, Lissette Cespedes, Regine Boutin, Mohammed Jaloudi, Maya Raghuwanshi

**Affiliations:** 1 Internal Medicine, Rutgers New Jersey Medical School, Newark, USA; 2 Endocrinology and Metabolism, Rutgers New Jersey Medical School, Newark, USA; 3 Hematology and Oncology, Rutgers New Jersey Medical School, Newark, USA

**Keywords:** thyroid, follicular thyroid cancer, papillary thyroid cancer, collision tumor

## Abstract

The term “collision tumor” is described as the coexistence of two or more histologically distinct neoplastic morphologies separated by normal tissue in the same organ. Simultaneous papillary thyroid carcinoma (PTC) and follicular thyroid carcinoma (FTC) of the same thyroid lobe is a very rare pathology. Herein, we report a case of PTC and FTC of the same thyroid lobe. A 79-year-old man was evaluated at our hospital for the presence of left hip pain of two-month duration after sustaining a physical trauma to the left side of his body three days prior to admission. X-ray imaging of the left femur revealed a large lytic bony lesion at the proximal end of left femur. Biopsy of the bone lesion was suggestive of FTC. Computed tomography (CT) of the neck revealed an enlarged thyroid with a cystic lesion in the left lobe of the thyroid gland. Total thyroidectomy was performed. Histopathology revealed two separate primary malignancies of PTC and FTC. Genetic studies for RAS gene mutation were negative. He was initiated on suppressive doses of levothyroxine following thyroidectomy. Three months after surgery, thyrotropin alfa stimulated 204.5 mCi I-131 was administered. At seven months of follow-up, the thyroglobulin level was in the lower end of the normal range and anti-thyroglobulin antibody (anti Tg) remained negative (< 1.0 IU/mL). He was doing well and reported no symptoms.

For each type of well-differentiated thyroid cancers, several genes have been identified. However, thus far, no specific gene mutation responsible for the pathogenesis of the different tumor types has been described. Management of thyroid collision tumor is usually complex due to the presence of different pathology in the tumor tissues and given the fact that literature on this condition is limited. Typically, the treatment needs to be individualized. Our report brings up a concept that the occurrence is a rare phenomenon of simultaneous mutation of different genes that could give rise to different thyroidal neoplasms.

## Introduction

Collision tumors are rare clinical entities wherein two histologically distinct tumor types occur at the same anatomic sites. Some of the organs implicated are the stomach, liver, adrenal gland, lungs, ovary, kidneys, and colon [1]. Regarding the thyroid gland, collision tumors are rare, constituting about 1% of all thyroid malignancies, with most literature describing co-occurring medullary thyroid carcinoma (MTC) and papillary thyroid carcinoma (PTC) [2,3]. Simultaneous PTC and follicular thyroid carcinoma (FTC) of the same thyroid lobe is a rare occurrence with limited clinical information. Herein, we report a case of PTC and FTC of the same thyroid lobe following a pathological fracture.

## Case presentation

A 79-year-old man presented to the emergency department for evaluation of left hip pain of two-month duration. Three days before presentation, he sustained a physical trauma to the left side of his body.

**Figure 1:**
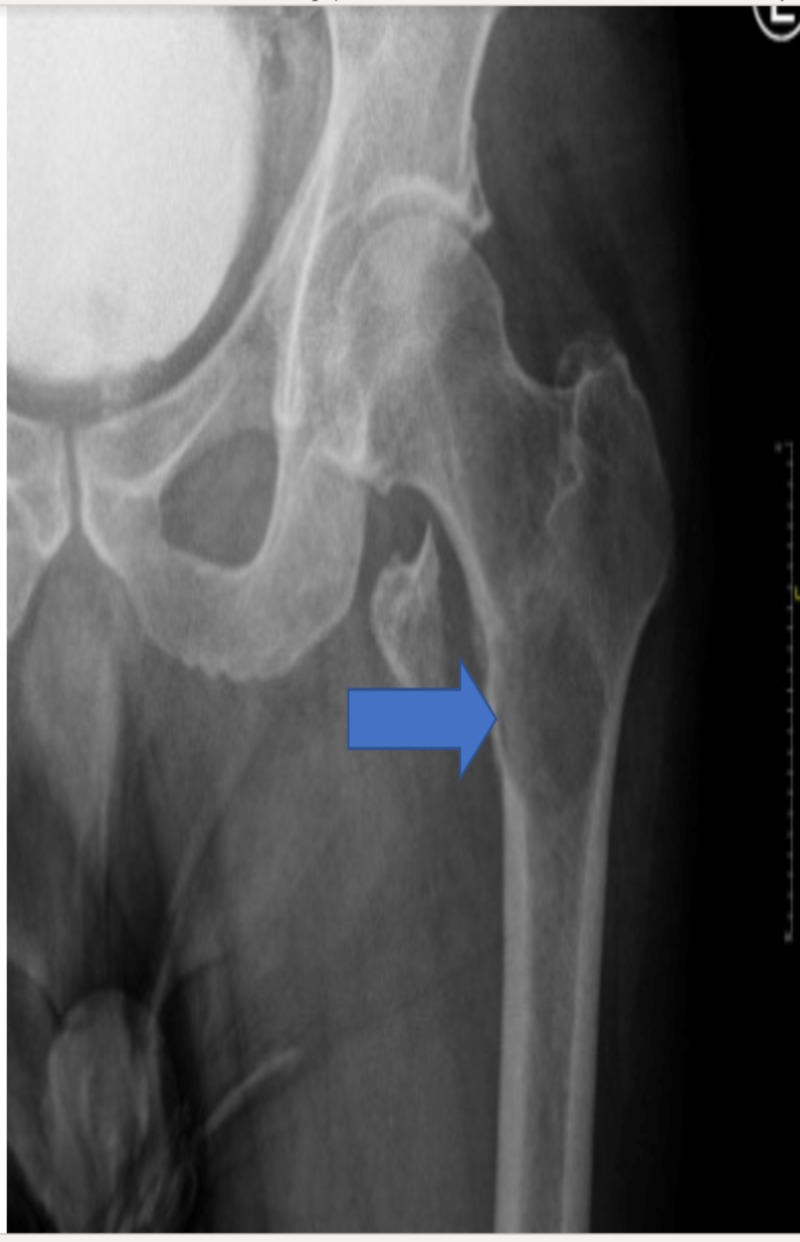
There is a lytic bony lesion measuring approximately 5.2 x 4.2 cm at the proximal left femur shaft extending to the intertrochanteric region and extending to the lesser trochanter; there is displaced pathological fracture of the left lesser trochanter

X-ray imaging of the left femur revealed a lytic bony lesion measuring approximately 5.2 cm x 4.2 cm at the proximal end of the left femur as well as a displaced pathologic fracture of its lesser trochanter (Figure 1).

**Figure 2:**
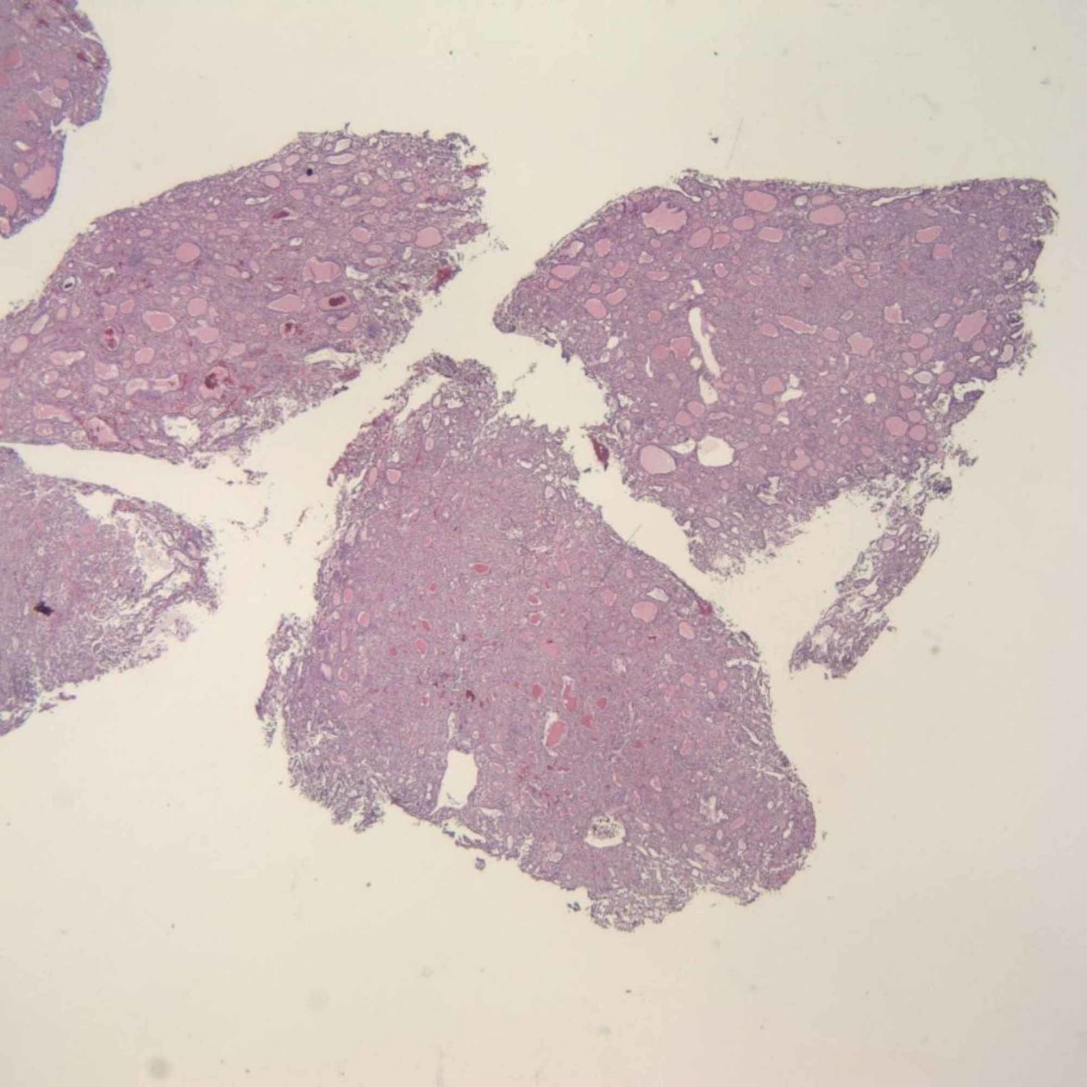
Metastatic follicular thyroid carcinoma (FTC) in the femur (hematoxylin and eosin stain, x40)

**Figure 3:**
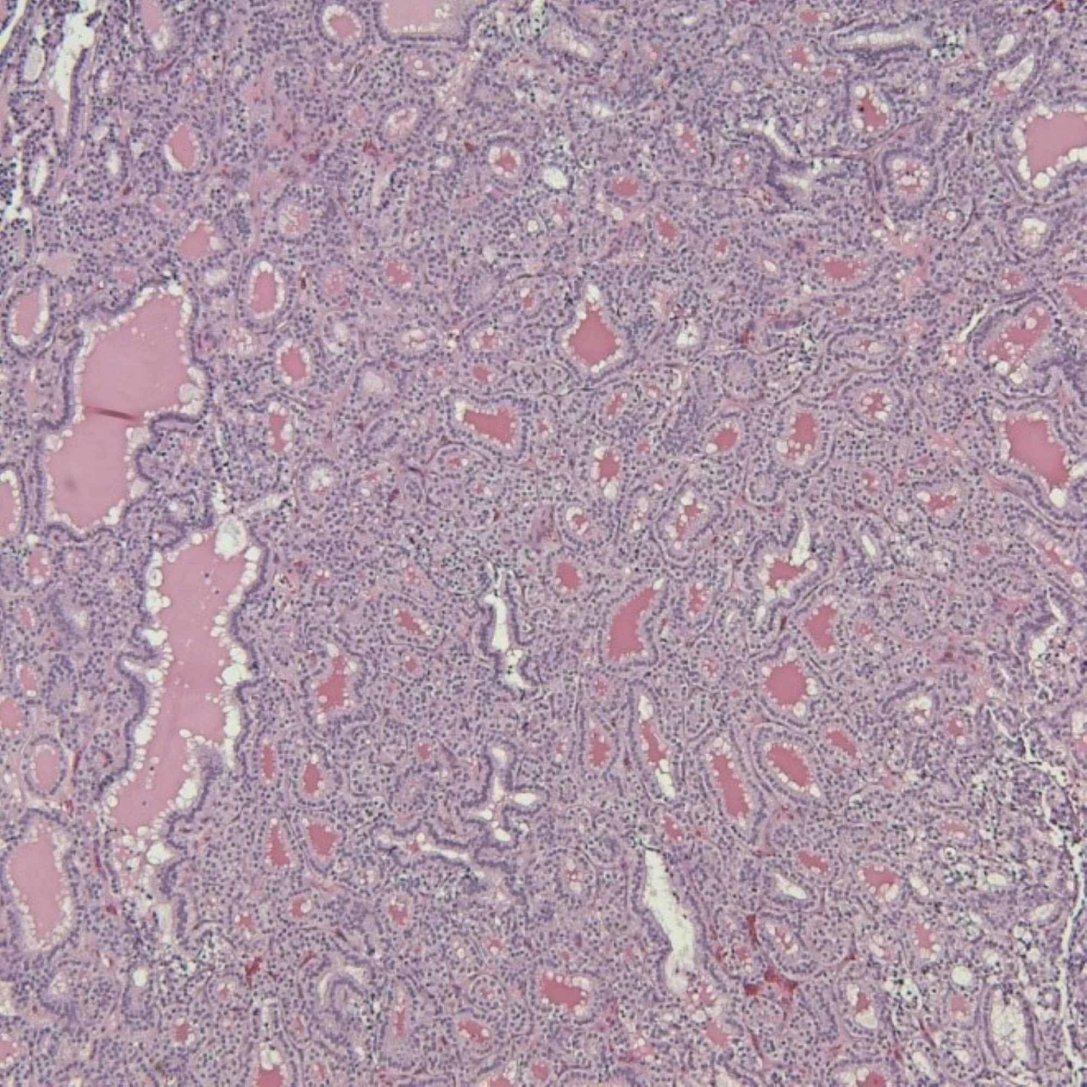
Metastatic follicular thyroid carcinoma (FTC) in the femur (hematoxylin and eosin stain, x100)

**Figure 4:**
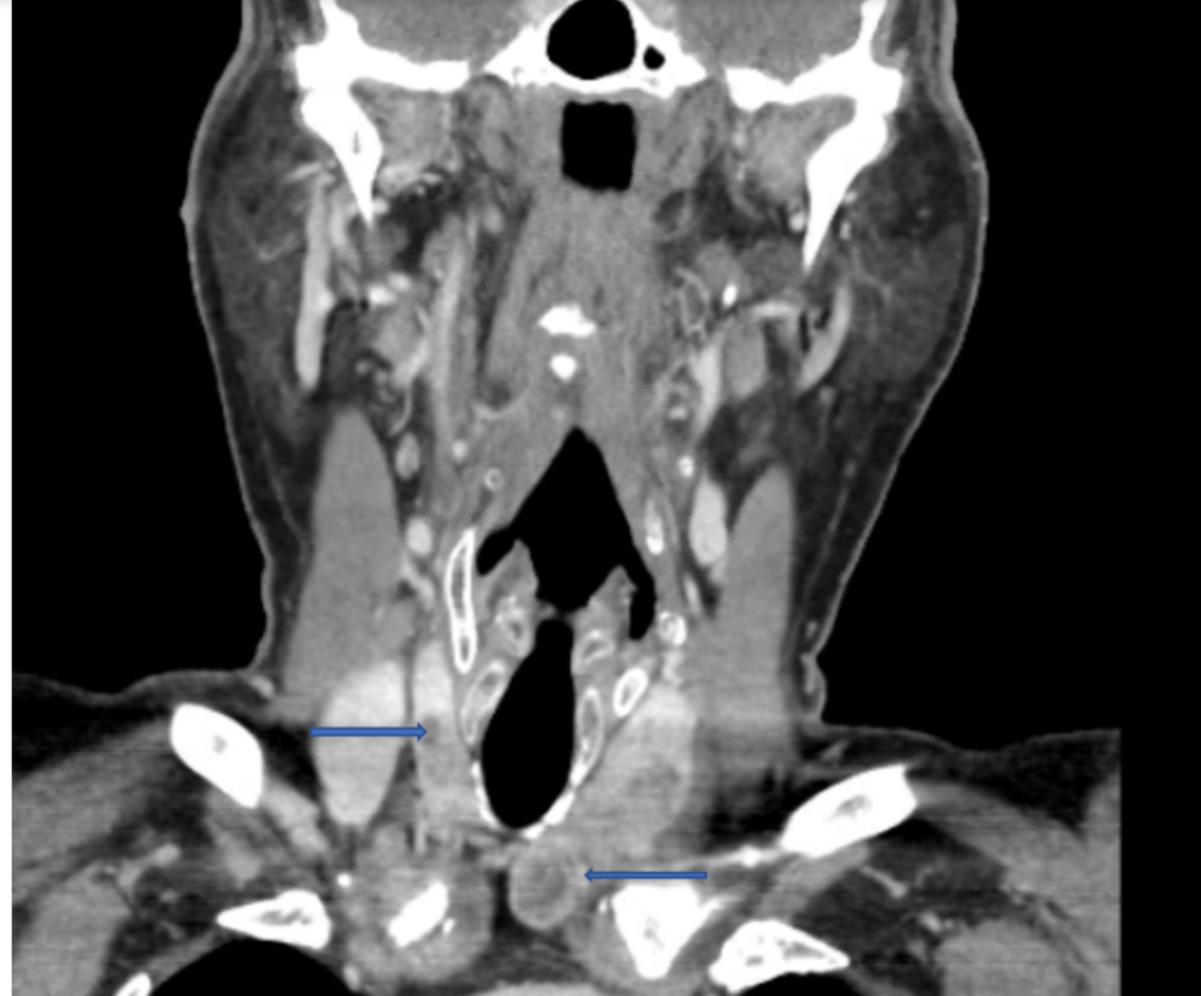
Hypoattenuating/hypoenhancing nodules seen in the right thyroid lobe measuring up to 7 mm, as well as a left thyroid mass with coarse calcifications, and a cystic region in its inferior portion

The patient underwent open reduction and internal fixation of the left femur. Biopsies of the bone lytic lesion suggested metastatic thyroid carcinoma, follicular type (Figures 2-3). Additional history was obtained. He had no history of radiation to the neck. There was no history of thyroid cancer in the family. Computed tomography (CT) of the neck revealed an enlarged thyroid with a cystic lesion as well as two nodules in the right lobe of thyroid gland (Figure 4). Ultrasound of neck was obtained which showed multiple nodules in both lobes of the thyroid including two right lobe complex nodules, spongiform composition measuring 0.7 x 0.8 x 0.7 cm, TR 1 and 0.6 x 0.6 x 0.5 cm, TR 1, as well as three left lobe solid, hypoechoic nodules measuring 2.9 x 3.6 x 2.5 cm, TR 3; 1.8 x 1.7 x 1.6 cm, TR 4 and 2.1 x 1.7 x 1.6 cm, TR 5.

**Figure 5:**
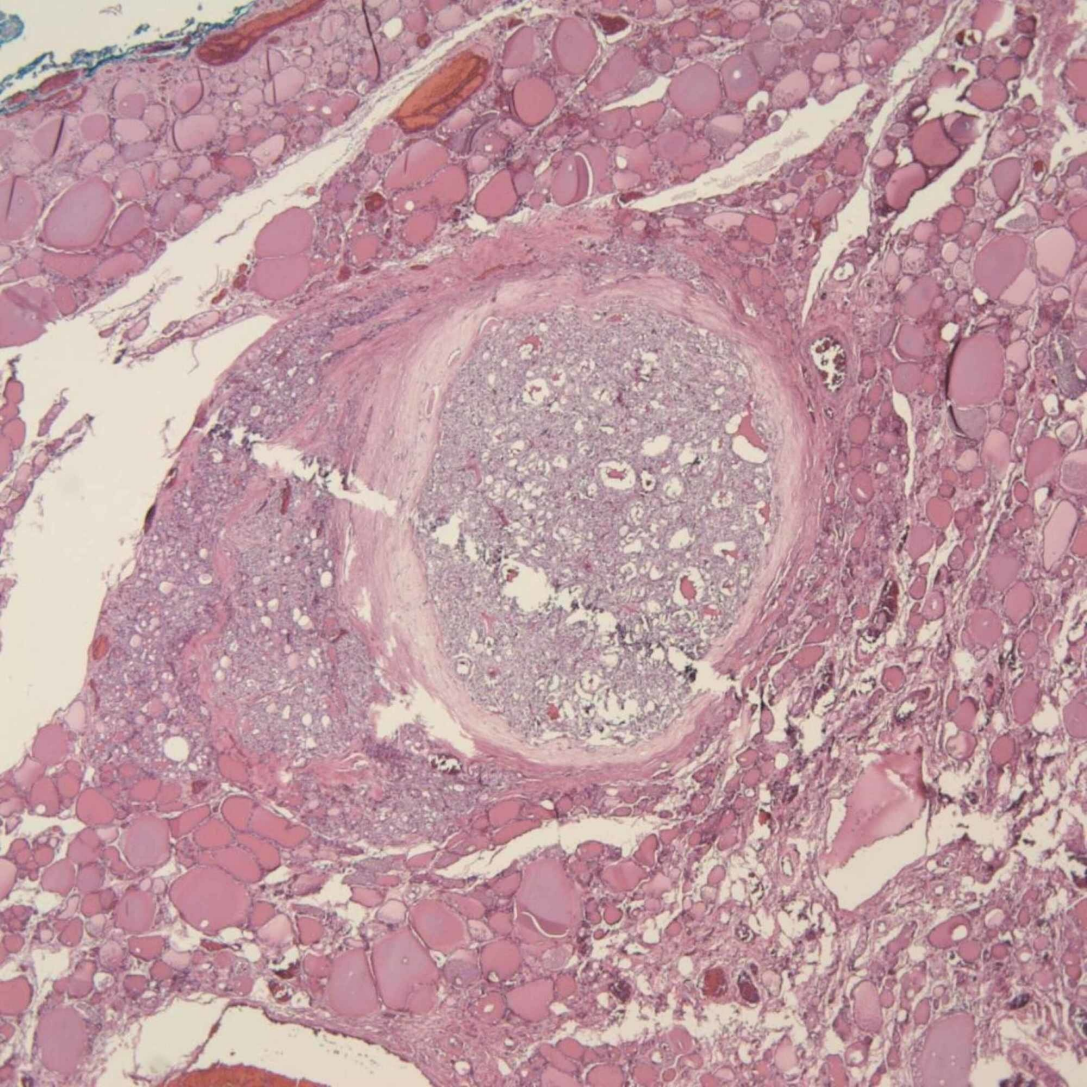
Papillary thyroid carcinoma (PTC) (hematoxylin and eosin stain, x40)

**Figure 6:**
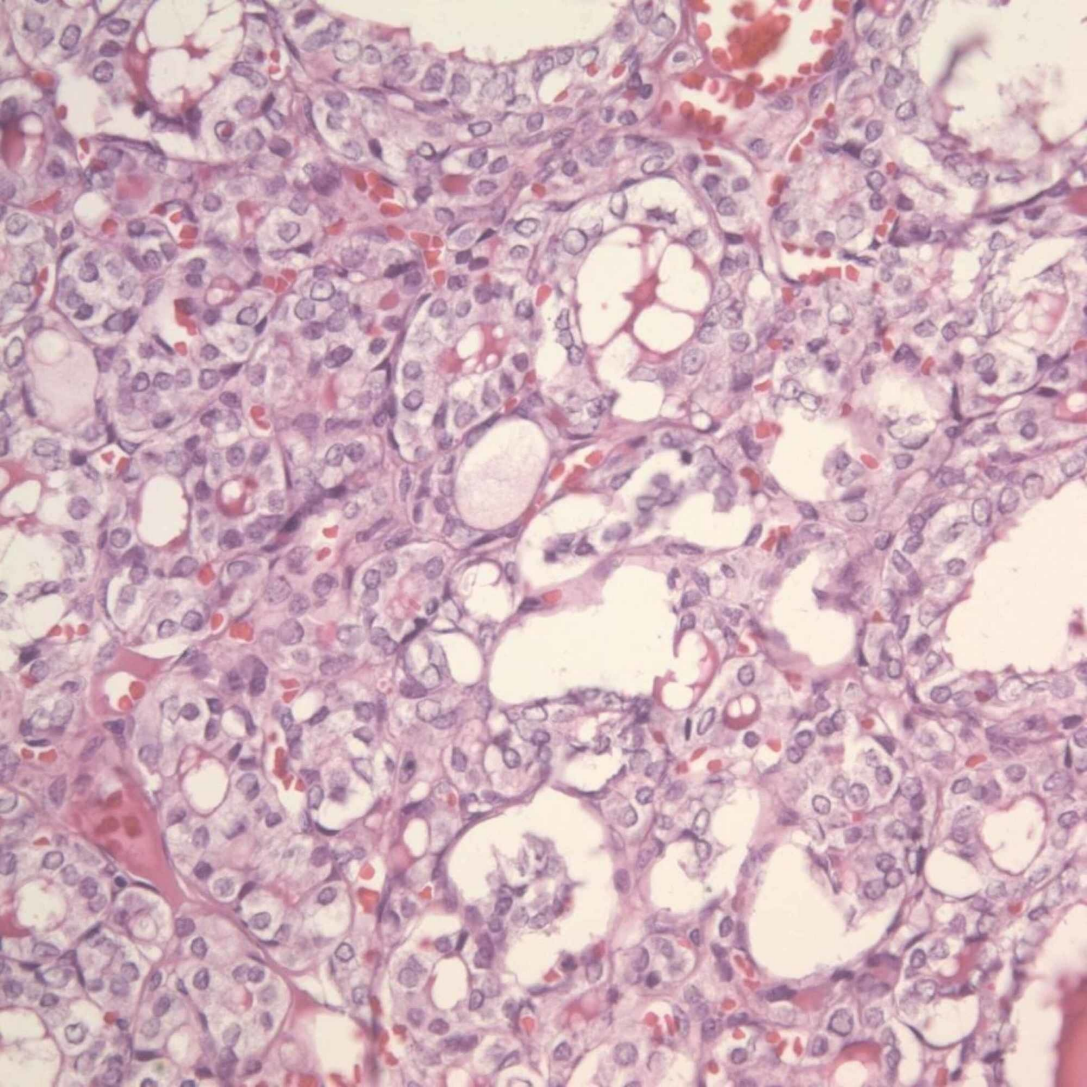
Papillary thyroid carcinoma (PTC) (hematoxylin and eosin stain, x40) showing nuclear features

**Figure 7:**
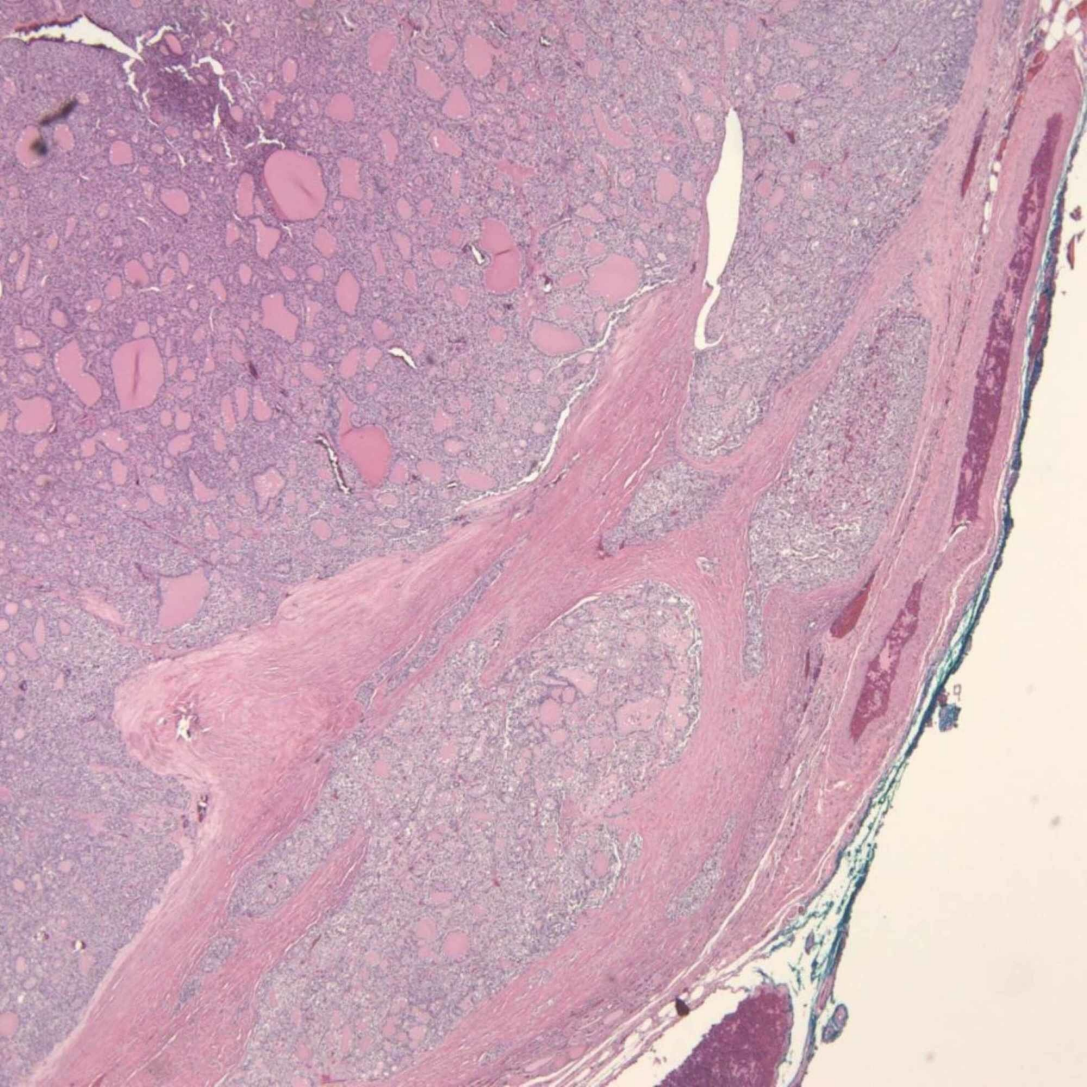
Follicular thyroid carcinoma (FTC) showing capsular invasion (hematoxylin and eosin stain, x40)

Total thyroidectomy was performed. Postoperative period of hospital stay was uneventful. Histopathology revealed two separate primary malignancies of PTC and FTC with extensive vascular invasion consistent with stage IVc T1bNXM1 disease. No lymphatic invasion or extrathyroidal extension was reported. (Figures 5-7).

**Figure 8:**
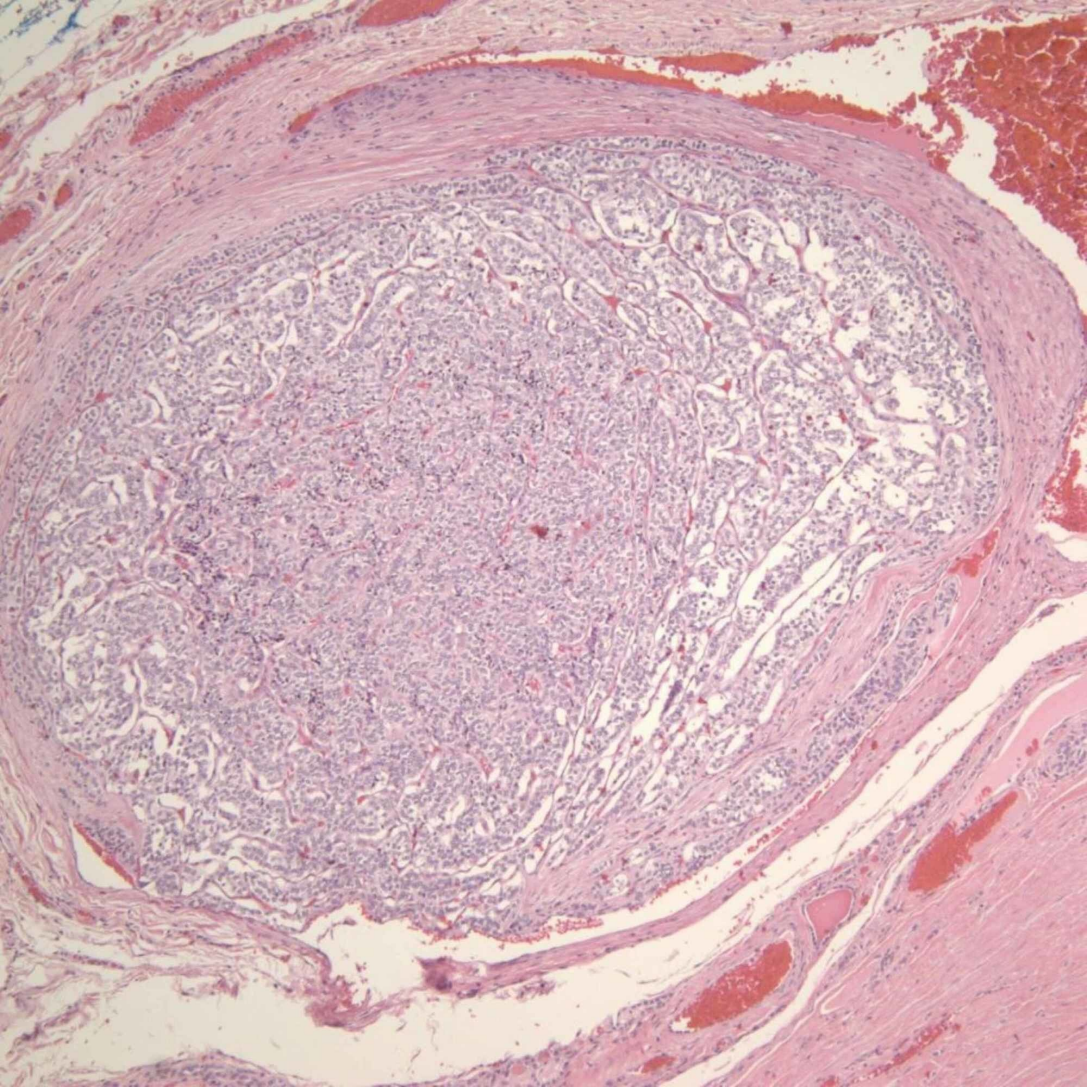
Follicular thyroid carcinoma (FTC) showing vascular invasion (hematoxylin and eosin stain, x100)

Following diagnosis, laboratory test results showed thyroid-stimulating hormone (TSH) 2.6 uIU/mL (reference range, 0.2-4), anti-thyroglobulin antibody (anti Tg) < 1.0 IU/mL (reference range, 0.0-0.9), calcitonin 8.4 pg/mL (reference range, 0-8.4), and carcinoembryonic antigen (CEA) 1.1 ng/mL (reference range, 0.0-3.0). Genetic studies for RAS gene mutation were negative. Two weeks after hip surgery, the patient began receiving a full course of external beam radiation to bone metastasis. He was also initiated on suppressive doses of thyroid hormone replacement therapy following thyroidectomy and the dose was subsequently increased to maintain TSH levels less than 0.1 uIU/mL.

Three months after initial presentation, thyrotropin alfa stimulated 204.5 mCi I-131 was administered. Thyroglobulin level was elevated at 76 ng/mL (reference range, 1.4-29.2) on the day of treatment. Subsequently, post-therapy whole body I-131 scan was performed which showed two focal areas of radioiodine uptake in the left proximal femur, consistent with metastatic bone disease. However, there was no evidence of other distant metastases.

At seven months of follow-up, thyroglobulin level declined to 2.5 ng/mL and anti Tg remained negative (< 1.0 IU/mL). He reported no pain on ambulation, dysphagia, choking sensation, neck pain, or hoarseness.

## Discussion

Thyroid carcinoma is the most common malignancy of the endocrine system and is one of the fastest growing cancers diagnosed in the Western world [4,5].

PTC is the most common malignancy of the thyroid, accounting for 70%-90% of thyroid cancers. FTC is the second most common type of malignancy affecting the thyroid, comprising about 5% of cases. FTC tends to spread by hematogenous routes leading to lung, bone, central nervous system metastases. Mortality rates associated with FTC are higher than for PTC, in part because a larger proportion of patients present with advanced stages of the disease. Poor prognostic features include age >50 years, distant metastases, primary tumor size >4 cm, presence of marked vascular invasion, and Hürthle cell histology [6-9].

Collision tumors of the thyroid gland are rare and present a diagnostic and treatment challenge. There is a paucity of reported literature on these tumors to date. There have been previous reports of simultaneous MTC, and PTCs reported in the literature. The first report of a collision tumor of FTC and PTC was made by Plauche et al. in the year 2013 [2].

No single theory can completely explain the pathogenesis of these tumors in all cases, and therefore, with the present level of understanding of the disease, a combination of theories should be considered.

The most widely accepted hypotheses suggested to explain the concurrent occurrence of MTC and FTC are random collision effect theory, stem cell theory, and hostage theory. The random collision effect theory suggests the possibility that two separate and distinct tumor types, either an MTC and an FTC or an MTC and a PTC, get initiated in close proximity to one another, thus resulting in a polyclonal neoplasm which, however, gets clinically recognized as a single tumor. The stem cell theory postulates that such tumors are the result of the ability of cancer stem cells to differentiate into different tumor cell lines in the same organ or anatomic site [1].

FTC and PTC are both types of differentiated thyroid carcinoma that generally carry a relatively good prognosis. However, it is thought that the presence of two primary cancers, even though of the differentiated type, could be signifying an aggressive behavior as well as an increased risk of recurrence [2].

For each type of thyroid malignancy, several genes have been identified. However, to date, no common gene mutation responsible for the pathogenesis of the different tumor types has been determined. For instance, point mutations of the RAS oncogene are found in about 40% of thyroid neoplasms (N-RAS, H-RAS, and K-RAS, in order of decreasing frequency) including both PTC and FTC [10-12]. The presence of RAS mutations in FTC appear to be associated with more aggressive cancers and higher mortality [13].

Management of collision tumors of the thyroid gland is usually complex owing to the presence of dual pathology in the tumor tissues and given the fact that literature on this condition is scarce. Ryan et al. suggested that treatment should be guided by the more aggressive of the neoplasms, while Sekar et al. recommended that each component of the combination should be treated like an independent primary malignancy [14,15]. Generally, the treatment needs to be individualized.

## Conclusions

There is a paucity of reported literature on collision tumors to date. No single theory can completely explain the pathogenesis of these tumors in all cases, and, therefore, a combination of theories should be accepted. As the knowledge of these rare tumors increase with greater reporting and follow‑up, particularly with respect to pathogenesis and treatment, it is hoped that, standardized diagnostic and treatment protocols will be evolved. Generally, patients need to be evaluated for molecular studies and genetic testing given the fact that, most likely, a rare phenomenon of simultaneous mutation of different genes can give birth to contemporary different thyroidal neoplasms. Management of collision tumors of the thyroid gland is usually complex and needs to be individualized.
